# Effects of sympathomodulation on pulse wave properties in heart failure with preserved ejection fraction

**DOI:** 10.1007/s00392-025-02798-y

**Published:** 2025-11-12

**Authors:** Luisa Koehler, Karl-Philipp Rommel, Paula Sagmeister, Maximilian von Roeder, Luise Mentzel, Holger Thiele, Philipp Lurz, Karl Fengler

**Affiliations:** 1 Department of Cardiology, Heart Center Leipzig at Leipzig University, Strümpellstr. 39, 04289 Leipzig, Germany; 2 Department of Cardiology, Cardiology I, University Medical Centre of the Johannes Gutenberg-University Mainz, Mainz, Germany

**Keywords:** Heart failure with preserved ejection fraction, Renal denervation, Interventional heart failure therapies, Pulse wave analyses

## Abstract

**Aims:**

One important contributor to the pathogenesis and perpetuation of HFpEF is unfavorable pulse wave reflection, frequently caused by increased arterial stiffness, due to aging and longstanding hypertension. One possible path to address pathological wave reflections is reducing sympathetic overactivity via renal denervation (RDN) with the aim to enhance arterial compliance. We sought to non-invasively quantify pulse wave reflections changes in patients undergoing RDN.

**Methods:**

Patients who underwent RDN for resistant hypertension were classified as patients with HFpEF and patients without heart failure, the latter of which served as the control group. Pulse wave analysis (PWA) was assessed using a piezoelectric device, and reflection magnitude was quantified by the augmentation index (AIx), and timing by backward transit time (BTT) before and 3 months after RDN. Ambulatory blood pressure was assessed at baseline and after 3 months to adjust for blood pressure changes.

**Results:**

Of 51 patients, 22 had HFpEF, and 29 served as controls. While baseline values in AIx and BTT were not different between HFpEF and control patients, RDN was associated with a significant decrease in AIx by 5.6% (*p* = 0.04) and a significant prolongation of BTT by 17.8 ms (*p* = 0.007) in patients with HFpEF. Conversely, no significant changes were observed in control patients for AIx (*p* = 0.13) and BTT (*p* = 0.56).

**Conclusions:**

The study observed a beneficial change in the timing and magnitude of central wave reflections in HFpEF after RDN. This implies a dynamic component of arterial stiffness in HFpEF patients and renders large artery stiffness a potential therapeutic target.

**Graphical Abstract:**

**Figure:**
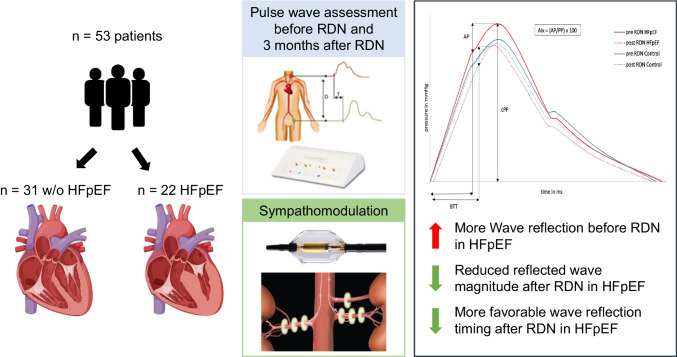

## Introduction

Heart failure with preserved ejection fraction (HFpEF) is an important contributor to morbidity and mortality worldwide [1, 2]. The high prevalence of this condition is contrasted by very limited treatment options beyond symptomatic therapy. HFpEF is characterized by a reduced exercise capacity [3]. Arterial stiffness, frequently associated with aging and prolonged hypertension, sets forth a chain of adverse consequences [4]:

The gradual loss of arterial compliance fosters heightened wave reflections, adding to the heart’s afterload [5]. Consequently, diastolic filling diminishes, elevating left ventricular end-diastolic pressure, and prompting myocardial hypertrophy—ultimately intensifying diastolic dysfunction, a distinctive HFpEF characteristic [6–10].

By addressing sympathetic overactivity through renal denervation (RDN), an avenue emerges to potentially modulate neurohormonal pathways implicated in vascular tone and arterial stiffness. The resulting decrease in sympathetic drive holds the potential to enhance arterial compliance and mitigate wave reflections, cumulatively contributing to a reduction in cardiac afterload among HFpEF patients in addition to RDN-induced blood pressure reduction [11].

Recently, we were able to show potentially beneficial effects of RDN on the pulsatile components of blood pressure using an indirect method from magnetic resonance imaging (MRI) and invasive pressure tracings [12]. While these data have provided valuable insights, the utilization of bedside tools bears the hope for scalability, as assessing the carotid–femoral pulse wave velocity presents a straightforward and economical method to assess the wave properties of cardiac output [13].

This study aims to investigate the effects of RDN on arterial stiffness in HFpEF patients by employing advanced non-invasive pulse wave analysis (PWA). We also seek to corroborate and extend the findings of our previous MRI study showing a reduction in pulse wave augmentation index (AIx) and backward transit time (BTT).

## Methods

We performed a predefined sub-study of a prospective study on the predictive value of arterial stiffness for the outcome of RDN patients. The predefined aim of this sub-study was to assess the changes in non-invasively assessed arterial stiffness after 3 months. The study was conducted at the Heart Center Leipzig at Leipzig University Center between June 2015 and May 2020 in accordance with the Declaration of Helsinki and after acceptance by a local ethics board [14].

### Patient selection

Patients underwent PWA using the Complior (Complior Analyse, Alam Medical, Saint Quentin Fallavier, France) device before and three months after the RDN procedure. Only patients with sufficient data quality and measurements at both time points were included in the analysis. Patients eligible for this study had a diagnosis of resistant hypertension, defined as systolic daytime ambulatory blood pressure measurement (ABPM) > 135 mmHg, despite being treated with at least three different classes of antihypertensive drugs, with each drug being administered at least 50% of the maximum dosage indicated for hypertension. It was mandatory for patients to be on at least one diuretic unless they were intolerant to diuretics. The antihypertensive medication regimen had to remain stable for a minimum of 4 weeks before enrollment. Medication was kept stable during follow-up in all patients.

Exclusion criteria for the study were as follows: patients under the age of 18 or over 75 years, pregnant individuals, those with a life expectancy of less than 6 months, evidence of untreated secondary hypertension, known renal artery stenosis, or anatomical unsuitability for interventional RDN. Furthermore, patients with a main renal artery diameter less than 4.0 mm were also excluded from the study [14].

### Group definition

Patients were divided into two groups: The first group comprises HFpEF patients based on the diagnostic criteria of elevated N-terminal pro-B-type natriuretic peptide (NT-pro BNP) blood levels > 125 pg/mL, NYHA functional class II or higher, and diastolic dysfunction on echocardiography, which includes left atrium (LA) size (volume index) > 35 mL/m^2^, mitral E velocity > 90 cm/s, septal e′ velocity < 9 cm/s, early filling velocity on transmitral Doppler/early relaxation velocity on tissue Doppler (E/e′) ratio > 9 and a preserved ejection fraction (EF) > 50. The second group comprised patients with normal EF and without heart failure, serving as controls. The grouping of the participants was based on the European Society of Cardiology heart failure guidelines of 2021 [15].

### Pulse wave analysis

Non-invasive PWA was performed using the Complior, a piezoelectric device [16]. The measurements were taken while the participants were in a supine position after a minimum of 5 min of rest. PWA measurements were taken by trained physicians.

The main parameters that are used to assess arterial stiffness and wave reflections are the augmentation index (AIx) and the carotid-femoral pulse wave velocity (cPWV). The AIx is expressed as a percentage and represents the proportion of the central pulse wave that is augmented by these reflections [17].

Measurement of cPWV involves simultaneous recordings of the arterial pulse waveforms at both the carotid and femoral arteries [18].

Other recorded parameters were central and peripheral systolic blood pressure (cSBP, pSBP) and central and peripheral diastolic blood pressure (cDBP, pDBP), the backward transit time (BTT) with correlated pressure (Pretro), the end-systolic pressure (PES), the left ventricular ejection time (LVET) and diastolic time (DT).

### RND procedure

Endovascular ultrasound renal denervation was conducted using the Paradise™ catheter (ReCor Medical, Palo Alto, CA, USA), a balloon-cooled device that utilizes acoustic energy to create a fully circumferential thermal ablation pattern [19–21]. A balloon of appropriate size was carefully inserted into the renal artery and precisely inflated under cine fluoroscopic guidance to ensure optimal contact with the renal artery wall. At each treatment site, ultrasound energy was delivered for 7 s, followed by deflation of the balloon. Lesions were strategically placed along the renal artery, starting from the distal and progressing toward the proximal portion. All procedures were performed by experienced interventional cardiologists with significant expertise in utilizing ultrasound for renal denervation. To manage visceral pain during the procedure, intravenous remifentanil was administered. The transfemoral access route was consistently employed for all patients.

### Statistical analysis

All statistical analyses were performed using appropriate software SPSS 29.0.1.0 (IBM, NY, USA). Descriptive statistics, including means, and standard deviations were calculated for continuous variables, while frequencies and percentages were determined for categorical variables. The clinical characteristics were analyzed using the Pearson Chi-square test.

Paired *t*-tests were used to compare normally distributed continuous parameters between baseline and follow-up or with non-parametric tests as appropriate. Adjustment for blood pressure changes was calculated using an analysis of covariance (ANCOVA) with blood pressure changes as a covariate.

A *p*-value of less than 0.05 was considered statistically significant.

## Results

### Baseline parameters

Of the 80 patients enrolled in the trial, a full data set at baseline and follow-up was available for 51 patients with normal ejection fraction. A total of 22 patients (43%) were allocated to the HFpEF group, while the remaining 29 (57%) patients were assigned to the control group. The baseline characteristics separated into HFpEF and non-HFpEF patients are presented in Table 1.

**Table 1:** Patient characteristics

	All (*n* = 51)	HFpEF (*n* = 22)	Control (*n* = 29)	*p*-value
Age (years)	64.1 ± 8.5	65.4 ± 8.0	63.1 ± 8.9	0.34
Female, *n* (%)	16 (31)	9 (41)	7 (24)	0.20
BMI (kg/m^2^)	31.2 ± 5.0	32.9 ± 5.4	29.8 ± 4.3	0.03
Smoker, *n* (%)	29 (58)	12 (55)	17 (55)	0.92
Diabetes, *n* (%)	23 (45)	14 (64)	9 (31)	0.02
History of myocardial infarction, *n* (%)	4 (8)	2 (9)	2 (7)	0.77
Peripheral arterial disease, *n* (%)	6 (11)	5 (23)	1 (3)	0.04
Hypercholesterolemia, *n* (%)	41 (78)	16 (73)	24 (83)	0.39
Coronary artery disease, *n* (%)	21 (41)	12 (55)	9 (31)	0.09
Atrial fibrillation, *n* (%)	4 (8)	2 (9)	2 (7)	0.77
nt-pro-BNP (pg/mL)	174 (76;327)	180 (95;515)	161 (55;319)	0.29
NYHA ≥ 2, *n* (%)	23 (45)	22 (100)	1 (3)	0.001
Central systolic blood pressure (mmHg)	159.5 ± 30.0	164.6 ± 37.3	155.7 ± 22.9	0.30
Central diastolic blood pressure (mmHg)	89.8 ± 14.3	90.4 ± 14.6	89.3 ± 14.3	0.80
Ambulatory systolic blood pressure (mmHg)	149.8 ± 12.8	151.9 ± 13.6	148.3 ± 12.3	0.32
Ambulatory diastolic blood pressure (mmHg)	84.8 ± 12.2	83.9 ± 12.4	85.4 ± 12.3	0.67
Central pulse pressure (mmHg)	69.9 ± 27.7	74.6 ± 36.4	66.3 ± 18.6	0.30
Augmentation index (%)	18.9 ± 16.5	18.0 ± 16.4	19.6 ± 16.8	0.74
Pulse wave velocity (m/s)	11.2 ± 3.2	11.5 ± 3.2	10.9 ± 3.2	0.52
Augmentation pressure (mmHg)	13.3 ± 14.3	12.5 ± 15.6	13.9 ± 13.4	0.74
Systolic time (ms)	201 ± 52	201 ± 61	202 ± 44	0.92
Backward transit time (ms)	107 ± 33	109 ± 31	105 ± 34	0.68
Retrograde pressure magnitude (mmHg)	144.3 ± 27.3	149.3 ± 34.2	140.5 ± 20.5	0.26
End-systolic pressure (mmHg)	123.3 ± 18.7	121.2 ± 20.7	124.5 ± 17.3	0.73
Left ventricular ejection time (ms)	323 ± 37	328 ± 40	320 ± 34	0.36
Deceleration time (ms)	629 ± 148	635 ± 182	625 ± 120	0.90
Heart rate (min^−1^)	66 ± 12	67 ± 14	64 ± 10	0.61
Ejection fraction (%)	62 ± 7	60 ± 7	63 ± 6	0.06

Data is presented as means and standard deviation or IQR

In the HFpEF patient group, diabetes (*p* = 0.02), higher BMI (*p* = 0.03), and peripheral arterial vascular disease (*p* = 0.04) were more prevalent. The other parameters did not show significant differences between the groups.

### Blood pressure

Systolic measurements of ABPM were significantly reduced at follow-up across the HFpEF group (151.9 ± 13.6 vs. 141.5 ± 14.7, *p* = 0.02) and the control group (148.3 ± 12.3 vs. 139.4 ± 12.4, *p* < 0.001). Diastolic blood pressure values were also reduced in the HFpEF group (83.9 ± 12.4 vs. 75.8 ± 12.8, *p* < 0.001) and the control group (85.4 ± 12.3 vs. 79.2 ± 10.3, *p* < 0.001). The systolic central blood pressure, in turn, exhibited no significant change in the HFpEF group (164.6 ± 37.3 vs. 154.7 ± 21.3 *p* = 0.26) and not in the control group (155.7 ± 22.9 vs. 155.7 ± 21.1, *p* = 0.99). Also, the diastolic central blood pressure did not show a significant alteration in the HFpEF group (90.4 ± 14.7 vs. 86.0 ± 16.2, *p* = 0.20) and the control group (89.3 ± 14.3 vs. 90.0 ± 12.3, *p* = 0.76, Table 2).

**Table 2:** Clinical changes and pulse wave analysis

	All (*n* = 51)		*p*-value (pre vs. post)	HFpEF (*n* = 22)		*p*-value (pre vs. post)	Control (*n* = 29)		*p*-value (pre vs. post)
	Baseline	Follow-up		Baseline	Follow-up		Baseline	Follow-up	
Nt-proBNP (pg/mL)	174 (76, 327)	151 (57, 373)	0.06	180 (95, 515)	177 (68, 422)	0.97	161 (55, 319)	104 (47, 311)	0.08
central systolic blood pressure (mmHg)	159.5 ± 30.0	155.3 ± 21.0	0.37	164.6 ± 37.3	154.7 ± 21.3	0.26	155.7 ± 22.9	155.7 ± 21.1	0.99
central diastolic blood pressure (mmHg)	89.8 ± 14.3	88.3 ± 14.1	0.42	90.4 ± 14.6	86.0 ± 16.2	0.20	89.3 ± 14.3	90.0 ± 12.3	0.76
systolic ambulatory blood pressure (mmHg)	149.8 ± 12.8	140.3 ± 13.3	< 0.001	151.9 ± 13.6	141.5 ± 14.7	0.02	148.3 ± 12.3	139.4 ± 12.4	< 0.001
diastolic ambulatory blood pressure (mmHg)	84.8 ± 12.2	77.8 ± 11.5	< 0.001	83.9 ± 12.4	75,8 ± 12,8	< 0.001	85.4 ± 12.3	79.2 ± 10.3	< 0.001
Central pulse pressure (mmHg)	69.9 ± 27.7	66.6 ± 23.3	0.44	74.6 ± 36.4	67.7 ± 24.5	0.39	66.3 ± 18.6	65.8 ± 22.7	0.90
Augmentation index (%)	18.9 ± 16.5	13.4 ± 16.5	0.02	18.0 ± 16.4	12.4 ± 15.1	0.04	19.6 ± 16.8	14.2 ± 17.7	0.13
Heart rate (min^−1^)	66 ± 12	65 ± 10	0.34	69 ± 14	68 ± 11	0.69	64 ± 10	63 ± 8	0.37
Pulse wave velocity (m/s)	11.2 ± 3.2	11.2 ± 2.7	0.94	11.5 ± 3.2	10.7 ± 2.4	0.11	10.9 ± 3.2	11.6 ± 2.9	0.32
Augmentation pressure (mmHg)	13.3 ± 14.3	8.2 ± 10.2	0.01	12.5 ± 15.7	8.5 ± 10,3	0.15	13.9 ± 13.4	8.1 ± 10.5	0.04
Systolic time (ms)	201 ± 52	190 ± 49	0.07	201 ± 61	186 ± 48	0.037	202 ± 44	193 ± 50	0.36
Backward transit time (ms)	107 ± 33	116 ± 35	0.08	109 ± 31	126 ± 34	0.007	105 ± 34	110 ± 35	0.56
Retrograde pressure magnitude (mmHg)	144.3 ± 27.3	144.4 ± 20.7	0.99	149.3 ± 34.2	143.7 ± 21.7	0.48	140.5 ± 20.5	144.8 ± 20.2	0.33
End-systolic pressure (mmHg)	123.3 ± 18.7	119.9 ± 15.4	0.18	121.2 ± 20.7	117.2 ± 16.3	0.32	124.5 ± 17.3	121.8 ± 14.7	0.38
Left ventricular ejection time (ms)	323 ± 37	319 ± 39	0.40	328 ± 40	321 ± 44	0.08	320 ± 34	318 ± 36	0.84
Deceleration time (ms)	629 ± 148	637 ± 169	0.77	635 ± 182	608 ± 153	0.31	625 ± 120	657 ± 179	0.42

Data is presented as means and standard deviation or IQR

### Pulse wave analysis

**Fig. 1:**
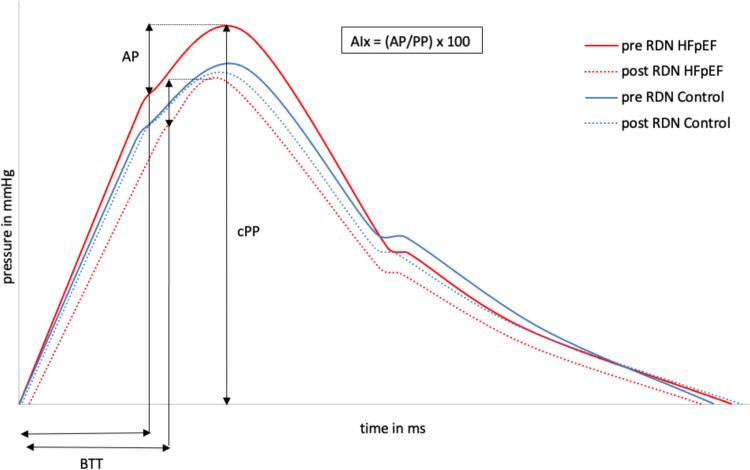
Schematic illustration of the central pulse wave pre-RND and 3-month post-RND in the HFpEF and control groups. The modification of the backward transit time (BTT) and the augmented pressure (AP), which represents the significant change in magnitude and timing of the pulse wave, is shown. Data is visualized as a pulse wave with average values for each parameter

**Fig. 2:**
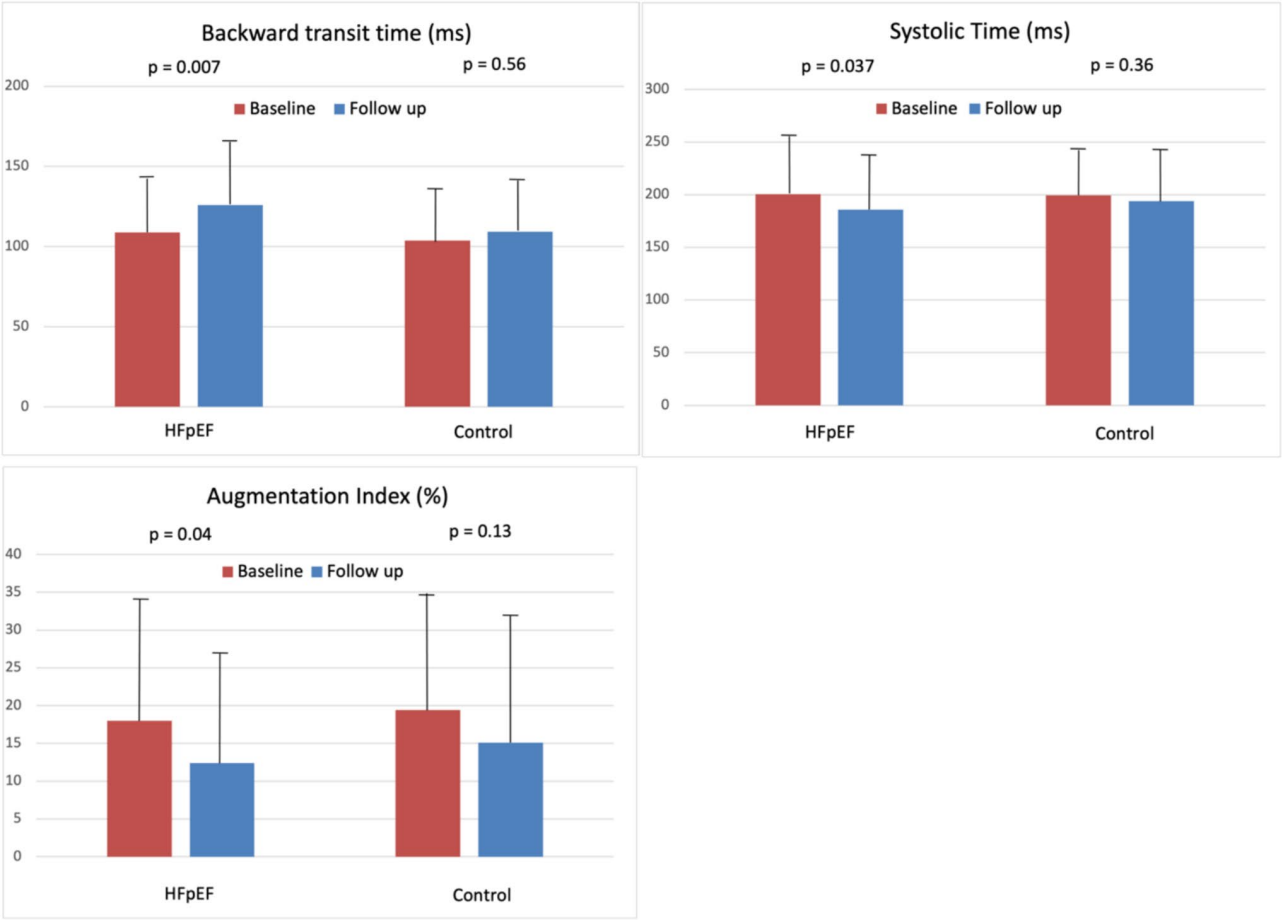
Changes in augmentation index, systolic time, and backward transit time pre-RND and 3-month post-RND in the HFpEF and control groups. Data is visualized as a histogram with standard deviation

At baseline, there was no significant difference between the groups related to pulse wave analysis parameters AIx (*p* = 0.74), AP (*p* = 0.74), cPP (*p* = 0.30), BTT (*p* = 0.68) and PWV (*p* = 0.52). In the HFpEF group, RDN was associated with a significant reduction in the AIx (18 ± 16.4 vs. 12.4 ± 15.1, *p* = 0.04) and a significant prolongation of the BTT (108.7 ± 30.8 vs. 125.77 ± 34, *p* = 0.007, Figs. 1 and 2) after 3 months. In the control group, no significant changes were observed for AIx (19.6 ± 16.8 vs. 14.2 ± 17.7. *p* = 0.21) and BTT (105 ± 34 vs. 110 ± 35, *p* = 0.56). After adjustment for blood pressure changes, the change in AIx in the HFpEF group was not significant while BTT remained significant (*p* = 0.33 and 0.01 respectively). The HFpEF group also showed a significant reduction of the systolic time (200.5 ± 61.3 vs. 185.6 ± 48.25, *p* = 0.037), while the control group did not (*p* = 0.36). The heart rate did not differ significantly in the HFpEF group (69 ± 14 vs. 68 ± 11, *p* = 0.69) and the control group (64 ± 10 vs. 63 ± 8, *p* = 0.17). There was no significant change in the PWV in the HFpEF group (11.5 ± 3.2 vs. 10.7 ± 2.4, *p* = 0.11) and the control group (10.9 ± 3.2 vs. 11.6 ± 2.9, *p* = 0.32). The AP significantly decreased in the control group after RDN (13.9 ± 13.4 vs. 8.1 ± 10.5, *p* = 0.04), but not in the HFpEF group (12.5 ± 15.7 vs. 8.6 ± 10.2, *p* = 0.15). Outcome parameters are represented in Table 2.

## Discussion

This is the first study attempting to investigate alterations in pulse wave properties among patients with arterial hypertension and HFpEF following RDN, utilizing non-invasive pulse wave analysis.

The hypothesis of wave property modulation with sympathomodulation finds support through the non-invasive pulse wave analysis employed in this investigation.

It is known that patients with HFpEF have a stiffer vascular system [22]. In our study, baseline stiffness parameters did not differ significantly between patients with HFpEF and controls. This may reflect the presence of a dynamic component in HFpEF, which becomes more evident under physiological stress or exertion [23].

In the current investigation, the reduction in AIx and the prolongation of retrograde wave time indicate a shift in the timing and magnitude of wave reflection, leading to a more favorable waveform. These alterations suggest a more favorable hemodynamic profile, potentially reducing the strain on the left ventricle. The observed phenomenon could be associated with an increased total impedance mismatch before RDN, considering a more pronounced sympathetic influence on arterial stiffness [24, 25].

In line with the hypothesis of a reduced afterload and improved ventriculo-arterial coupling, a reduction of left ventricular mass has been described following RDN [26].

In combination with other vascular changes after RDN, like improved aortic distensibility and reduced markers of left ventricular (LV) filling pressures, hemodynamic and vascular alterations after RDN translate into improved heart-failure symptoms: New York Heart Association functional class was significantly reduced after RDN in a cohort of 164 patients with and without HFpEF [24].

The reduction in blood pressure, a notable outcome of RDN, adds an intriguing dimension to its impact on HFpEF. This reduction may contribute synergistically to the altered pulse wave characteristics, potentially influencing LV afterload and, consequently, ventricular loading [24]. Nevertheless, adjustment for blood pressure changes suggests blood pressure reduction as the main mechanism of changes in the reflected wave’s magnitude. Also, as AP was reduced in the control group, these changes might also be a direct consequence of blood pressure reduction.

Interestingly, this was not observed for BTT. This suggests additional blood pressure–independent, sympathetic mediated mechanisms in wave propagation and reflection.

The biomechanical adjustment reflects an enhancement in arterial compliance and distensibility. The resultant effect of this biomechanical shift manifests in a recalibrated temporal interplay between cardiac systole and the propagation of arterial pressure waves, thereby optimizing ventricular–vascular coupling and ameliorating cardiac afterload. This adaptive response aligns with an ameliorated AIx, signifying an attenuation of wave reflections and ventricular workload, which collectively underscore the salient role of decreased arterial stiffness in orchestrating enhanced cardiac performance through optimization of ventricular–vascular interaction [12, 24, 27].

However, the exact mechanisms behind these alterations remain elusive, necessitating further exploration.

Previous studies showed inconclusive effects of RDN on PWV, the parameter considered the most important marker for arterial vascular stiffness [28]. One study on 94 patients found a small reduction of PWV after RDN (10.0 vs. 9.8 m/s) using a non-invasive technique [29], while a study from our own group did not find a reduction of invasively assessed PWV [30]. In the current study, we also did not observe a significant reduction of PWV, even though there was a numerical reduction. As RDN leads to a reduction of systemic sympathetic activity, a reduction of the modifiable, vasomotoric component of PWV is overall likely. The lack of a statistically significant effect might be explained by the relatively small sample sizes in our previous invasive study (23 patients) as well as in the current study. As PWV poses an important risk factor for cardiovascular events and its reduction might translate into improved cardiovascular outcomes, investigating the effects of RDN on PWV warrants further investigation.

### Limitations

The current study has some limitations. Firstly, there is a potential impact of subclinical stenotic conditions and adipose tissue on the accuracy of measurements. Stenosed arteries may induce irregular waveforms, distorting the acquired data and leading to potential inaccuracies in arterial stiffness assessment. Moreover, the presence of substantial adipose tissue within the measurement site can dampen the pressure wave, affecting the fidelity of PWA results.

Secondly, the study cohort is a convenience sample, potentially introducing selection bias. Thirdly, the overall sample size is small, and the potential for identifying a notable array of statistically significant outcomes within our study is attenuated. All observations herein warrant further confirmation, even though overall results seem to align with our previous MRI-based study. Also, more extensive statistical analyses as well as adjustment for various potential confounders were not feasible due to the small sample size and large dispersion. Moreover, the distinction between HFpEF and non-HFpEF patients remains difficult, as clinical, echocardiographic pulse wave findings often overlap.

### Clinical implications

The study shows a significant change in the timing and magnitude of wave reflection after RDN toward a more favorable waveform, imposing less strain on the left ventricle in HFpEF patients. This could enhance diastolic heart function, potentially alleviating HFpEF symptoms and improving patients’ quality of life. This insight might guide personalized RDN candidacy, optimizing its benefits.

These observations suggest a significant, reversible element of arterial malfunction in HFpEF, encouraging additional investigations.

## Data Availability

The data that support the findings of this study are available from the corresponding author upon reasonable request.

## Abbreviations

ABPM: Ambulatory blood pressure monitoring
AIx: Augmentation index
AP: Augmented pressure
BP: Blood pressure
BTT: Backward transit time
cPWV: Carotid-femoral pulse wave velocity
DBP: Diastolic blood pressure
E/e′ ratio: Early filling velocity on transmitral Doppler/early relaxation velocity on tissue Doppler
HFmrEF: Heart failure with mildly reduced ejection fraction
HFpEF: Heart failure with preserved ejection fraction
HFrEF: Heart failure with rejected ejection fraction
LA: Left atrium
LV: Left ventricular
LVET: Left ventricular ejection time
NT-pro BNP: N-terminal pro-B-type natriuretic peptide
PES: End-systolic pressure
PP: Pulse pressure
PWA: Pulse wave analysis
RDN: Renal denervation
SB: Systolic blood pressure
